# Collection of Recombinant Rotaviruses Expressing Fluorescent Reporter Proteins

**DOI:** 10.1128/MRA.00523-19

**Published:** 2019-07-03

**Authors:** Asha A. Philip, Brittany E. Herrin, Maximiliano L. Garcia, Andrew T. Abad, Sarah P. Katen, John T. Patton

**Affiliations:** a Department of Biology, Indiana University, Bloomington, Indiana, USA; KU Leuven

## Abstract

A collection of recombinant rotaviruses that express the fluorescent markers UnaG, mKate, mRuby, TagBFP, CFP, or YFP as separate proteins was generated. Genes for the fluorescent proteins were inserted into genome segment 7 without compromising expression of the protein NSP3. These recombinant rotaviruses are valuable for analyzing rotavirus biology by fluorescence-based live-cell imaging.

## ANNOUNCEMENT

**FIG 1:**
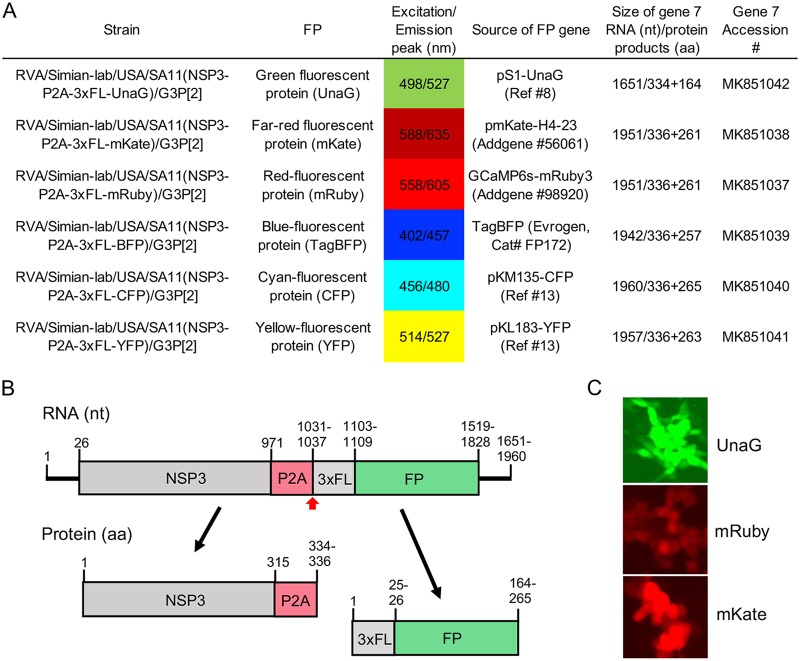
Recombinant SA11 rotavirus expressing fluorescent reporter proteins. (A) Formal names of SA11 strains producing FPs, properties and sources of the FPs, and sizes of the modified gene 7 (NSP3) RNA and protein products that direct FP expression. GenBank accession numbers are provided for the modified gene 7 RNAs (https://www.ncbi.nlm.nih.gov/genbank/) (13). (B) Schematics of the modified gene 7 RNAs and protein products for SA11 strains expressing FPs. The RNA schematic includes coding positions for NSP3, porcine teschovirus 2A-like (P2A) element, 3×FLAG tag, and FP and indicates the position of the stop-restart position in the P2A element (red arrowhead). The protein schematic reflects the two protein products generated by the activity of the P2A element. (C) Fluorescence detected at 8 h postinfection (p.i.) in MA104 cells infected with recombinant SA11 rotaviruses expressing UnaG, mRuby, or mKate using a Zoe (×20) fluorescent cell imager (Bio-Rad).

Rotaviruses are major causes of severe, potentially life-threatening gastroenteritis in young children (1). Like other members of the *Reoviridae* family, rotaviruses have a segmented double-stranded RNA (dsRNA) genome that is replicated using viral positive-sense RNA [(+)RNA] as a template. Recently, an efficient plasmid-based reverse genetics (RG) system was developed that allows any of the 11 dsRNA segments of the rotavirus genome (strain SA11) to be genetically modified (2–4). As tools for analyzing rotavirus biology, we used a modified RG system to produce recombinant rotaviruses that express fluorescent reporter proteins (FPs) (5–7). These viruses were engineered to express the FPs as separate proteins without deleting or interrupting any of the open reading frames (ORFs) in the viral genome that direct viral protein synthesis. Through this effort, we succeeded in generating well-replicating recombinant SA11 rotaviruses that efficiently express UnaG (green), mKate (far-red), mRuby (red), TagBFP (blue), CFP (cyan), or YFP (yellow) (Fig. 1).

To generate the recombinant rotaviruses, we transfected baby hamster kidney cells expressing T7 RNA polymerase (BHK-T7) with 11 T7 (pT7) expression plasmids, each directing synthesis of a different viral (+)RNA, and a cytomegalovirus (CMV) plasmid that mediates expression of the RNA capping enzyme of African swine fever virus (ASFV) NP868R (8). The pT7 plasmid containing the segment 7 cDNA (pT7/NSP3) encodes NSP3, a moderately expressed viral protein that enhances translation of viral (+)RNAs and suppresses translation of host mRNAs (9). We modified the NSP3 ORF in the pT7/NSP3 plasmid, replacing it with an ORF encoding NSP3 fused downstream to a FLAG-tagged FP. To promote the expression of NSP3 and FPs as two separate proteins, we inserted a porcine teschovirus 2A-like (P2A) stop-restart translational element between the NSP3 and FLAG-tagged FP coding sequences (10, 11) (Fig. 1). Three days following transfection with RG plasmids, BHK-T7 cells were overseeded with simian MA104 cells. After an additional 4 days, clarified lysates were prepared from the cocultivated cells, and recombinant rotaviruses were isolated by plaque purification (12). Viruses containing FP sequences were identified by RNA gel electrophoresis and sequencing. Live-cell fluorescence imaging demonstrated that the recombinant viruses directed readily visible levels of fluorescent reporter proteins (Fig. 1).

This collection of recombinant rotaviruses provides a foundation for establishing fluorescence-based live-cell imaging methods for determining titers of the virus and monitoring spread of the virus, characterizing neutralizing antibodies and antiviral agents, and analyzing viral gene expression during the replication cycle. Moreover, rotaviruses expressing different FPs allow experiments designed to understand parameters affecting coinfection frequencies and the formation of reassortment viruses.

### Data availability.

The recombinant rotavirus strains in this collection are available to laboratories operating with biosafety level 2 (BSL2) certification that have received institutional approval to receive and perform experiments with rotavirus. Requests for strains should be directed to the corresponding author. GenBank accession numbers of the segment 7 RNAs in the rotavirus-2A/FP strains are given in Fig. 1.
